# Statistical analysis of bitcoin during explosive behavior periods

**DOI:** 10.1371/journal.pone.0213919

**Published:** 2019-03-22

**Authors:** José Antonio Núñez, Mario I. Contreras-Valdez, Carlos A. Franco-Ruiz

**Affiliations:** Department of Finance, Tecnologico de Monterrey, EGADE Business School, Mexico City, CDMX, Mexico; Nanyang Technological University, SINGAPORE

## Abstract

This paper develops the ability of the normal inverse Gaussian distribution (NIG) to fit the returns of bitcoin (BTC). As the first cryptocurrency created, the behavior of this new asset is characterized by great volatility. The lack of a proper definition or classification under existing theory exacerbates this property in such a way that explosive periods followed by a rapid decline have been observed along the series, meaning bubble episodes. By detecting the periods in which a bubble rises and collapses, it is possible to study the statistical properties of such segments. In particular, adjusting a theoretical distribution may help to determine better strategies to hedge against these episodes. The NIG is an appropriate candidate not only because of its heavy-tailed property but also because it has been proven to be closed under convolution, a characteristic that can be implemented to measure multivariate value at risk. Using data on the price of BTC with respect to seven of the main global currencies, the NIG was able to fit every time segment despite the bubble behavior. In the out-of-sample tests, the NIG was proven to have an adjustment similar to that of a generalized hyperbolic (GH) distribution. This result could serve as a starting point for future studies regarding the statistical properties of cryptocurrencies as well as their multivariate distributions.

## Introduction

The dawn of the XXI century has been characterized by high technological development by technology permeating through different areas of human interaction. One of these implementations is known as cryptocurrencies, which is term under which different financial assets are categorized. Originally intended as a substitute for the fiat money issued by central banks, these assets rely on peer-to-peer networking and mathematical cryptography to ensure their value [1]. Even when there is no government or financial institution that recognizes cryptocurrencies as a means of payment for goods or services, many of them have seen an increase in demand in recent years [2]. In particular, bitcoin (BTC) is considered the most important cryptocurrency, being the first to be created and having nearly 40% of the total cryptocurrency market cap on May 15^th^, 2018.

BTC´s creation dates back to the 2008 financial crisis. At that time, public sentiment tended toward discontent about how the economy was being directed and the impact that political decisions were having on the wellbeing of citizens. In this context, BTC was able to capitalize on the social unrest and loss of trust in governmental institutions and be adopted by many as an alternative to conventional money [3]. Created in 2009 by Satoshi Nakamoto, BTC consists of three parts: miners, the blockchain, and the wallet [4]. The first is a group of individuals who use cryptography to obtain one unit of BTC. This process is intended to replicate the effort of metal extraction and thereby prevent double spending. This newly extracted unit is then identified as a member of the blockchain, which guarantees that it cannot by copied, and then it may be transferred to a wallet. This mechanism is intended to be transparent and to avoid any discretionary expansion in the supply of BTC. Because of this process, the price of cryptocurrencies depends only on the demand side [5], an aspect that has created great volatility in this type of asset [6].

One fundamental aspect of cryptocurrencies in general, and BTC in particular, is the difficulties that emerge when trying to identify them within existing economic theory. By definition, fiat money is an asset recognized by a government or institution–a central bank–as a legal tender to pay for good, services, and taxes [7], and it is a store for value. This property is identified in the central bank´s balance sheet, which places issued money as part of its liabilities. As mentioned earlier, BTC is not universally recognized or backed by any government nor can it be used to buy assets of the real economy, so it cannot be categorized as such. Another point to highlight is that because of the high volatility of cryptocurrencies since their creation, they are unable to store value as other currencies do [8].

Other categories in which BTC may fit are gold-backed digital currencies or even commodities. For the first, the main problem arises when analyzing the underlying asset; even though the so-called mining process replicates metal extraction, it does not create any tangible element. Considering this aspect, BTC shares some characteristics with fiat money, where trust and the universal acceptance of value are key elements [1]. Moving to the commodity definition, according to the Cornell Law School, under the US Code, General Provisions, Chapter 1, § 1ª –Definition (9) states that commodities are material goods as well as services, rights, and interests. However, as mentioned earlier, there are no material goods behind this asset. It can be used to purchase certain services online but does not constitute a service *per se*. Finally, a right or interest needs to be recognized as such by a central authority. Thus Buchholz [3] considers these new entities to be something in between the previously mentioned definitions, which may help to explain the abrupt changes in price and ultimately lead to the appearance of bubbles.

Financial bubbles are defined by Phillips [9] as a rapid increase in price that diverges from the fundamental value or equilibrium. This explosive behavior eventually becomes unsustainable, causing the price to collapse, which is a property shared by all bubbles [10]. Given these considerations, Caginalp *et al*. [11] state that this type of episode is exacerbated by the poor availability of information or a lack of understanding about the assets that are being traded. As an example of this circumstance, they mention the case of internet companies being overvalued and causing a bubble in the market. However, as Andersen and Sornette [12] mention, the real problem becomes the identification of these deviations, as most of them are observable until the bubble bursts; nevertheless, the origin and duration of the deviation are difficult to determine. Taking these considerations into account, it is important not only to study the statistical properties of cryptocurrencies as a whole but also to conduct a focused analysis of the periods of explosive behavior.

Regarding the presence of heavy tail behavior, some authors have studied the stylized facts of the BTC and other cryptocurrencies. As an outstanding example, Bariviera *et al*. [13] study the behavior of the BTC in the period from 2011 to 2017 for daily data and in the period 2013 to 2016 for intraday data. In the case of daily data, the standard deviation of the BTC is approximately ten times greater than the standard deviation of the British pound (GBP) or the euro (EUR), and the distributions are non-normal. The BTC presents the property of long-range correlations, but according to the Hurst exponent of the GBP and EUR, these currencies behave according to the efficient market hypothesis. This last behavior of the three assets is similar when examining intraday frequency. With respect to intraday frequency, the price of the BTC has extreme changes and does not reaching a zone of stabilization (in 2013 and 2014), and the variance presents a diminishing trend. Alvarez-Ramirez *et al*. [14] studied the period 2013–2017 and showed that for BTC returns, there is asymmetry in the measure of correlations depending on whether the period is characterized by an increasing or a decreasing price. Important deviations from efficiency in different time scales are observed, and returns show anti-persistency over long periods. At the same time, they showed the existence of a fat tail where the negative tail was the heaviest.

In Zhang W. *et al*. [15], different stylized facts are investigated for eight cryptocurrencies, and the authors identified the existence of heavy tails, volatility clustering, long-range dependence, and zero autocorrelations for the returns.

In this context, the main study object is the returns of the asset, and thus the model and fit of a theoretical distribution become the main issue. As mentioned earlier, bubble periods may be distinguished by so-called explosive behavior and then an eventual collapse in price. Extrapolating this behavior to returns implies the presence of heavy tails in the distribution. Nevertheless, some additional considerations must be taken into account; according to Cont [16], financial assets also present skewness and excess kurtosis, meaning that it is necessary to propose a theoretical distribution flexible enough to capture these factors.

In particular, the distributions under the generalized hyperbolic (GH) family are determined by five parameters [17] that manage to fit the empirical distributions of many assets. This property is used by authors such as Eberlein and Keller [18], Eberlein and Prause [19] and Rydberg [20], and its first application to the field of finance is in modeling the returns of underlying assets in the US and German stock markets. Nevertheless, the normal inverse Gaussian distribution (NIG), a member of the GH Family, manages to capture the heavy tail behavior better [21]. In this case, financial assets that present this property in particular can be modeled with the NIG, which performs well in empirical applications such as valuation methods and volatility modeling [22]. For the study object of this work, this quality of the NIG becomes fundamental, as explosive movements and rapid downward movements identify bubble episodes.

Other studies addressing the NIG prove its ability to fit stocks [23] and index returns [24] as well as commodities such as gold and other precious metals [25]. As stated earlier, cryptocurrencies are financial assets that hold some similarities to other entities in how they are defined. As the NIG has proven to fit these objects, which are characterized by volatility and, in many cases, are underlying assets for other goods–just like cryptocurrencies–then it becomes a good candidate for adjusting BTC returns.

In this paper, we intend to prove the ability of the NIG to fit the returns of BTC even during bubble periods for seven exchange rates. Although some studies [26] state that a GH distribution is the best candidate to fit the data, the author suggests that it is the flexibility of the five parameters that generate the statistical criteria providing that result. In this sense, the NIG adjustment presents several advantages with respect to the GH one; in particular, it has praiseworthy mathematical properties as follows: 1) its functional expression is tractable, i.e., it is a close formula that can be easily worked with; 2) it is closed under affine transformations; 3) because it is fully determined by 4 parameters, the numerical process to obtain them is computationally easier because linked the stochastic process becomes deterministic, 4) the NIG has been proved to be the only member of the GH family to be closed under convolution [14], i.e., the sum of the NIGs is also an NIG; 5) in the data analysis, the daily series for the two major currencies in which the asset is traded are divided according to the bubble presence criteria, providing a stress test for particularly extreme value behavior in returns; and finally, 6) considering the regulatory criteria, the Basel Committee on Banking Supervision [27] state that the market risk must be evaluated under 95% values, which is a quantity fulfilled by the proposed distribution. In sum, if the hypothesis is accepted, it could prove that the NIG is sufficient to capture the high volatility of an asset as atypical as BTC; in conjunction, the trade-off between the marginal improvement in the adjustment of the GH is exceeded by the mathematical and computational advantages of the NIG, making it a more creditable candidate for evaluating and modeling BTC in practice. To do so, a goodness-of-fit test is implemented for each of the periods, followed by an out-of-sample value at risk (VaR) and expected shortfall (CVaR) comparison of NIG and GH. The importance of this result relapse in the mentioned NIG properties could lead to improved risk valuation and administration. Ultimately, it could provide a better understanding of explosive periods as well as mechanisms for hedging against this type of asset. The structure of the present work is as follows: section 2 presents the literature review; then, in section 3, the methodology is discussed, and finally, sections 4 and 5 respectively display the results and conclusions.

## Literature review

The literature on bubble detection has seen much development since the events of 2001, when the so-called dot-com bubble burst. One of these papers is that presented by Caginalp *et al*. [11], who expose a theoretical framework in which the current price should fluctuate around a fundamental value so that any positive divergence could eventually lead to bubble behavior. Relative to internet enterprises, they mention that even when traders have all the available information, decisions made by others operate as a factor that updates expectations. This behavior eventually leads to an overvaluation that becomes unsustainable in the long run, causing the critical readjustment to the equilibrium [28].

Considering that characteristic, Phillips *et al*. [9] present an analysis using NASDAQ information compared to declarations made by the then Chair of the Federal Reserve, Greenspan, about the existence of bubbles in that market. To do so, they present an econometric methodology based on forwarding recursive regression and a right-sided unit root test. Employing data from 1973 to 2005, they not only identify the presence of explosive behavior in prices but also detect the start and collapse of the bubble. Using this same methodology, Phillips *et al*. [29] analyze the real estate, commodity and bond markets during the 2008–2009 financial crisis. In this paper, they observe that the bubble began in the housing market and then spread by contagion to certain commodities and the bond market after the crisis entered the public domain. In this article, they also suggest that this methodology could be used as a to diagnose the market in order to prevent a financial and economic crisis.

Following these studies, Phillips *et al*. [30] propose a new methodology that is capable of detecting multiple bubbles in the time series. Starting from their previous work, in which they employ a right-sided unit root test via an augmented Dickey-Fuller (ADF) test, they propose the so-called generalized sup ADF or GSADF. By changing the amplitude of the window and using a recursive regression method, they manage to detect the origin and collapse of multiple bubbles in the same series. To test the model, S&P 500 data are employed from 1871 to 2010. In such analysis, the known bubble episodes are properly detected, ranging from the postwar boom in 1954 to the 1990 stock bubble.

Another application of this methodology is proposed by Alcock *et al*. [31], who aim to prove the existence of bubbles in the Australian market. To do so, they use the S&P ASX 200 data from 1992 to 2016 and apply different bubble detection models. By comparing these models, they conclude that the GSADF methodology is the most suitable for detection in price series.

Examining the presence of bubbles in BTC, Cheah and Fry [32] expose a fall of nearly 60% from the peak value of the cryptocurrency as an initial indication of the existence of this phenomenon. In their paper, they aim to prove not only that BTC is characterized by explosive behavior but also that it deviates from the fundamental value. By deploying economic models inspired by physics, they determine that the equilibrium value of BTC is zero. This result, as stated by the authors, could be a sign of speculative bubbles, and it could explain the high volatility observed since the creation of the cryptocurrency.

In a subsequent work, Fry and Cheah [33] expand their previous thesis to include the other most-traded cryptocurrency, Ripple. With an econophysics model and statistical analysis, the authors observe a negative impact on the prices of the analyzed assets. In this case, the presence of an increase in the value of Ripple exacerbates the drop in BTC price. This result could be interpreted as a competitive scenario in which the incorporation of new elements could lead to important changes in BTC’s market behavior.

The market efficiency hypothesis established by Fama [34] has been studied in the cryptocurrency sector. Kristoufek [35] states that the BTC time series against the dollar and yuan are inefficient in the period from 2010 to 2017, except in periods showing a collapse of BTC’s bubble-like behavior. Nadarajah and Chu [36] show for the first time that the weak efficiency of the market hypothesis is accomplished by a transformation of the odd integer powers of BTC price returns without any loss of information for the period from 2010 to 2016.

Kristoufek [37] contributes to the analysis of cryptocurrencies by pointing out the dominant factors in BTC price; through a wavelet coherence analysis, he defines a new perspective incorporating the relationships between the time and frequency of economic, transactional, technical, public interest and safe haven asset drivers, among others, and also investigates the impact of the Chinese market. Likewise, Garcia *et al*. [38] and Yelowitz and Wilson [39] outline the characteristics of BTC around socioeconomic and Google trends, respectively.

Zheng-Zheng *et al*. [40] study the behavior of BTC using Phillips’ GSADF model to detect the periods in which bubble episodes occur. In this case, the authors use the price of BTC relative to the US dollar (USD) and the Chinese yuan (RMB), as a discrepancy can be observed among the nominal exchange rates. These authors use a series from June 16^th^, 2011, to September 18^th^, 2017, with weekly periodicity. Their results show the presence of six bubbles in the Chinese market and five in the American market. For the RMB, two bubbles can be detected in 2013: the first one lasting from February 7^th^ to May 2^nd^ and the other lasting from October 24^th^ to December 12^th^. The next four are observed from 2015 to 2017; that in 2015 is the smallest in duration and occurs at the end of October. In 2016, the bubble starts on December 22^nd^ and lasts until January 5^th^, 2017. The next bubble began on May 11^th^, 2017, and collapsed on June 29^th^, while the last one detected occurred on July 27^th^. In comparison, the USD price saw a small bubble in August 2012. The next difference could be seen in 2015, where there was no bubble; finally, the last two episodes in China correspond to only a single episode in America.

As mentioned earlier, the main characteristic of a bubble episode is the asset’s divergence from its fundamental value; a positive bubble eventually suffers a collapse. This behavior is mainly explained through the price variable; nevertheless, it can be approached by considering the return series. Some of the existing studies rely on a distribution analysis of those returns. Cont [16] develops a statistical study of the behavior of financial assets, detecting the need for theoretical distributions to have at least 4 parameters to gain sufficient flexibility to capture properties such as an excess of kurtosis, a negative skew, and a heavy tail.

In his seminal paper of 1977, Barndorff-Nielsen proposes the GH family (GH) [17]. This set of theoretical distributions are defined under five parameters, a factor that becomes their major advantage in modeling diverse events. In this same study, the normal inverse Gaussian distribution (NIG) is presented; by defining the lambda parameter as equal to -0.5, the close formula is obtained. Deeper work on this formula proved some key features highlighted here: the main one is the ability to fit heavy tails in distributions, and the other is the property of being closed under convolutions, meaning that it is possible to sum NIGs and obtain the same NIG with different parameters. This property has been used to calculate the VaR of portfolios under the normality assumption; however, this hypothesis cannot be fully employed for financial assets.

The flexibility of the GH in fitting non-normal data has attracted interest in financial studies; in particular, Eberlein and Keller [18] use data from the DAX index to fit the theoretical GH distribution. In their study, they use data from 1989 to 1992 and conclude that the GH provides a better approximation for modeling financial returns than the normality assumption. However, Barndorff‐Nielsen [41] eventually conclude that the NIG is a better option for modeling financial returns.

In subsequent works, Trejo *et al*. [23] use data from the Mexican and American markets to fit the NIG. In their results, they conclude that members of the GH family are better candidates for fitting stock returns for those markets, leading to the conclusion that stochastic processes such as Brownian motion, in which normality is assumed, are not the best option for simulating financial series. On this topic, Nuñez *et al*. [24] prove the ability of the NIG to fit the indexes of BRIC economies even for periods of great volatility, such as during the crisis of 2008.

Moving to the commodities area, Shen *et al*. [25] discuss the ability of different members of the GH family to model returns on gold and other precious metals. These studies offer a proposal to improve the VaR calculus so it could be more precise, particularly as regards the left-sided heavy tail.

The application of the GH family to the returns of cryptocurrencies was originally proposed by Joerg [42]. In his paper, he offers an initial treatment of a statistical analysis of BTC and six other virtual currencies. In this study, the data are taken starting in 2016, and his conclusion is that returns of these new assets present heavy tails, so members of the GH family are the best suited to fit the observable data. Parallel to this work, Bueno *et al*. [6] use the complete series of BTC and fit the members of the GH family to obtain a better approximation of the VaR.

Considering the fit of log returns, Chu *et al*. [26] use the USD exchange rate relative to BTC to test for a better GH distribution that fits the series. For this study, they employ data from September 13^th^ 2011, to 8^th^ March 2014. For an initial test using descriptive statistics, they note the differences in the exchange rates of diverse currencies; they highlight features such as the smaller minimum, the larger mean and maximum, and the wider range. For the distribution comparisons, they test for 15 theoretical distributions that are members of exponential and hyperbolic generalized families. Using the maximum likelihood criteria, they conclude that the weakest distribution in terms of its match to the empirical results is the normal, while the best is the GH.

## Methodology

For the statistical analysis proposed by this paper, the results achieved by Zheng-Zhen *et al*. [40] are used to select the periods in which bubble episodes occurred. As shown by the authors, it is necessary to study BTC series relative to the USD and RMB. Nevertheless, to expand the analysis toward the major world currencies, other exchange rates are included, such as the Great Britain pound (GBP), the Hong Kong dollar (HKD), the Japanese yen (JPY), the Brazilian real (BRL) and the euro (EUR). The selection of these currencies was made in accordance with the data availability as well as the presence of a trading market. The data are obtained from Investing, https://www.investing.com/, and cover from July 7^th^, 2015, to May 16^th^, 2018, for the in-sample adjustments.

To study the different behaviors of the data during the bubble periods, the complete series was partitioned into periods. Intervals were then selected to contain one bubble episode each. The observations for the out-of-the-sample tests correspond to prices from May 17^th^, 2018, to January 14^th^, 2019. The exact dates for the in-sample periods are shown in Table 1.

**Table 1:** Dates from selected periods.

Period	Dates	Obs.
**1**	07/07/2015–31/05/2016	330
**2**	01/06/2016–01/05/2017	300
**3**	02/05/2017–16/05/2018	380

The dates were chosen according to the results of Zheng-Zheng (2018) with the daily periodicity found in Investing.

To appropriately investigate the series, the first test implemented aimed to ascertain the presence of a unitary root, i.e., whether the price series are stationary. To determine this, the ADF statistic, which is constructed under the null hypothesis of the series having a unitary root, was calculated. The results obtained for the raw prices of the series are shown in Table 2, while the logarithmic returns are in Table 3.

**Table 2:** Augmented Dickey-Fuller test for prices.

Period	RMB	GBP	HKD	JPY	BRL	EUR	USD
**1**	0.3758	0.2932	0.317	0.416	0.4785	0.3803	0.2862
**2**	0.4877	0.394	0.5417	0.4428	0.5291	0.4638	0.7383
**3**	0.7554	0.7429	0.7614	0.7681	0.7955	0.8156	0.8167

The p-values of the test. Data are from Investing and elaborated by the authors.

**Table 3:** Augmented Dickey-Fuller test for returns.

Period	RMB	GBP	HKD	JPY	BRL	EUR	USD
**1**	0.01	0.01	0.01	0.01	0.01	0.01	0.01
**2**	0.01	0.01	0.01	0.01	0.01	0.01	0.01
**3**	0.01	0.01	0.01	0.01	0.01	0.01	0.01

The p-values of the test. Data are from Investing and elaborated by the authors.

With these results, it is possible to confirm that the original price series for all of the currencies are nonstationary. Nevertheless, for the first difference, it is clear that the series becomes integrated order zero (I (0)). The reason to do so is presented by Campbell *et al*. [43]: as returns are scale-free, the different exchange rates can be turned into a common basis. The second reason is the ergodicity property, which guarantees the extension of the model to future periods. Therefore, for the statistical analysis, the returns rather than the price of BTC will be used.

To obtain the returns of the series, the continuous approach was used as follows:

$$r_{i}=lnP_{i}-lnP_{i-1}\;\forall i\epsilon T$$

such that r is the logarithmic return, and P is the price in the ith period.

To test the ability of this formulation to use empirical data, a diverse methodology is taken into account. First, the descriptive statistics of each series in the time segments are presented, followed by a statistical test for non-normality to confirm the possibility of adjusting the NIG. Once the parameters are obtained, a goodness-of-fit test is employed, so the results have statistical robustness. With these results, it can be confirmed that NIG can be used in the return analysis and simulation even in the presence of extreme value episodes. Finally, as an additional test, the VaR and CVaR are obtained for the out-of-sample data. These statistics are presented for normal, NIG and GH distributions to compare the results from empirical data.

### Descriptive statistics and normality test

To obtain a first indication for the use of a heavy-tailed distribution, descriptive statistics were obtained. As shown in Tables 4–10, it is possible to observe the same properties described for financial series: a non-zero skew and an excess of kurtosis. It is possible to observe the high levels in the series for all periods, so heavy-tailed behavior can be expected.

**Table 4:** Descriptive statistics for BTC/RMB.

Period	Mean	Variance	Skew	Kurtosis^a^
**1**	0.002610582	0.001432361	-0.2157669	6.324952
**2**	0.00331773	0.001382751	-0.6823923	3.087805
**3**	0.004499845	0.003494038	-0.3777803	3.925958

Data are from Investing and elaborated by the authors

^a^The excess of kurtosis

**Table 5:** Descriptive statistics for BTC/GBP.

Period	Mean	Variance	Skew	Kurtosis^a^
**1**	0.002591943	0.001571095	-0.07021559	8.18224
**2**	0.003661356	0.001460913	-0.530964	3.60333
**3**	0.004578335	0.003393543	-0.3685531	3.893634

Data are from Investing and elaborated by the authors

^a^The excess of kurtosis

**Table 6:** Descriptive statistics for BTC/HKD.

Period	Mean	Variance	Skew	Kurtosis^a^
**1**	0.002416748	0.001629951	0.04209298	8.828451
**2**	0.003162901	0.001394517	-0.6394187	2.897495
**3**	0.004748211	0.003582342	-0.4298761	4.068716

Data are from Investing and elaborated by the authors

^a^The excess of kurtosis

**Table 7:** Descriptive statistics for BTC/JPY.

Period	Mean	Variance	Skew	Kurtosis^a^
**1**	0.002059424	0.001526931	-0.1842234	7.49358
**2**	0.003149787	0.001381624	-0.7724884	3.348675
**3**	0.004673094	0.003637457	-0.2140974	4.01502

Data are from Investing and elaborated by the authors

^a^The excess of kurtosis

**Table 8:** Descriptive statistics for BTC/BRL.

Period	Mean	Variance	Skew	Kurtosis^a^
**1**	0.002481365	0.0006378073	0.4843842	5.571529
**2**	0.002760418	0.0009499151	-0.9525216	7.638152
**3**	0.005017262	0.002772499	0.3263481	6.726957

Data are from Investing and elaborated by the authors

^a^The excess of kurtosis

**Table 9:** Descriptive statistics for BTC/EUR.

Period	Mean	Variance	Skew	Kurtosis^a^
**1**	0.002021439	0.0007367653	-0.5953886	6.303397
**2**	0.003504393	0.001141889	-1.043421	6.290746
**3**	0.004417921	0.00284999	-0.02791827	1.826545

Data are from Investing and elaborated by the authors

^a^The excess of kurtosis

**Table 10:** Descriptive statistics for BTC/USD.

Period	Mean	Variance	Skew	Kurtosis^a^
**1**	0.001994404	0.001330794	-2.228013	29.21603
**2**	0.003701893	0.0009752682	-1.046945	4.72087
**3**	0.004456062	0.003127462	-0.05445456	1.626952

Data are from Investing and elaborated by the authors

^a^The excess of kurtosis

For the normality test, four criteria were chosen: Anderson-Darling, Shapiro-Francia, Lilliefors and Cramér-von Mises. These statistics operate under the null hypothesis that samples come from the same distribution, in this case, the Gaussian. The results of these are presented in Tables 11–17. By analyzing the p-values of the test, the null hypothesis is rejected, so the alternative, in which the sample does not come from the Gaussian distribution, is not rejected under a 99% confidence level.

**Table 11:** Normality test for BTC/RMB.

Period	Anderson-Darling	Shapiro-Francia	Lilliefors	Jarque-Bera	Cramér-von Mises
**1**	2.2e-16	2.638e-13	2.712e-11	2.2e-16	7.37e-10
**2**	3.8e-11	8.142e-09	1.768e-05	2.2e-16	2.657e-08
**3**	1.566e-12	4.071e-10	3.525e-07	2.2e-16	4.078e-09

Values are the p-values of the statistical tests for normality, elaborated by the authors.

**Table 12:** Normality test for BTC/GBP.

Period	Anderson-Darling	Shapiro-Francia	Lilliefors	Jarque-Bera	Cramér-von Mises
**1**	2.2e-16	1.579e-14	5.362e-11	2.2e-16	7.37e-10
**2**	2.36e-11	2.509e-09	3.505e-07	2.2e-16	3.117e-08
**3**	9.911e-11	7.795e-10	4.936e-05	2.2e-16	2.568e-08

Values are the p-values of the statistical tests for normality, elaborated by the authors.

**Table 13:** Normality test for BTC/HKD.

Period	Anderson-Darling	Shapiro-Francia	Lilliefors	Jarque-Bera	Cramér-von Mises
**1**	2.2e-16	3.299e-15	8.756e-16	7.37e-10	2.2e-16
**2**	1.281e-09	3.627e-08	3.687e-06	2.2e-16	1.386e-07
**3**	1.142e-11	3.719e-10	2.033e-06	2.2e-16	1.105e-08

Values are the p-values of the statistical tests for normality, elaborated by the authors.

**Table 14:** Normality test for BTC/JPY.

Period	Anderson-Darling	Shapiro-Francia	Lilliefors	Jarque-Bera	Cramér-von Mises
**1**	2.2e-16	4.044e-14	1.256e-13	2.2e-16	7.37e-10
**2**	1.625e-09	8.388e-09	2.067e-07	2.2e-16	3.153e-07
**3**	2.989e-11	5.142e-10	5.132e-06	2.2e-16	1.643e-08

Values are the p-values of the statistical tests for normality, elaborated by the authors.

**Table 15:** Normality test for BTC/BRL.

Period	Anderson-Darling	Shapiro-Francia	Lilliefors	Jarque-Bera	Cramér-von Mises
**1**	1.011e-13	5.733e-11	1.035e-06	2.2e-16	2.486e-09
**2**	2.2e-16	2.725e-13	4.864e-11	2.2e-16	7.37e-10
**3**	2.2e-16	1.901e-12	4.85e-10	2.2e-16	7.37e-10

Values are the p-values of the statistical tests for normality, elaborated by the authors

**Table 16:** Normality test for BTC/EUR.

Period	Anderson-Darling	Shapiro-Francia	Lilliefors	Jarque-Bera	Cramér-von Mises
**1**	2.2e-16	6.177e-13	2.646e-11	2.2e-16	7.37e-10
**2**	2.2e-16	3.904e-15	2.2e-16	2.2e-16	7.37e-10
**3**	2.414e-05	7.965e-06	0.001679	1.57e-12	0.0001269

Values are the p-values of the statistical tests for normality, elaborated by the authors.

**Table 17:** Normality test for BTC/USD.

Period	Anderson-Darling	Shapiro-Francia	Lilliefors	Jarque-Bera	Cramér-von Mises
**1**	2.2e-16	2.2e-16	2.2e-16	2.2e-16	7.37e-10
**2**	2.2e-16	7.891e-13	2.2e-16	2.2e-16	7.37e-10
**3**	0.0003209	5.705e-05	0.007178	3.825e-10	0.0008138

Values are the p-values of the statistical tests for normality, elaborated by the authors

Once non-normality is confirmed and using descriptive statistics, it is possible to consider the use of NIG to fit the data in the selected periods.

### Theoretical framework

The NIG was mathematically defined by Barndorff‐Nielsen in 1977 [14] by:

$$g(x;\alpha ,\beta ,\mu ,\delta )=a(\alpha ,\beta ,\mu ,\delta )q{\left(\frac{x-\mu }{\delta }\right)}^{-1}K_{1}\left\{\delta \alpha q(\frac{x-\mu }{\delta })\right\}exp(\beta x)$$

where:

$$a(\alpha ,\beta ,\mu ,\delta )=\pi^{-1}\alpha \;\exp\left(\delta \sqrt{\alpha^{2}-\beta^{2}}\right)-\beta \mu )$$

where:

$$q(x)=\sqrt{1+x^{2}}$$

such that K_1_ is the modified Bessel function of third order, and index 1 and α, β, μ and δ are parameters that satisfy 0≤ β ≤α,μ ϵ R and 0<δ. Under this parameterization, α represents the flatness of the density, meaning the concentration of values around the mean μ; β defines the skew level; and δ represents the scale of it. There exist multiple parameterizations for this distribution; nevertheless, the one proposed in this paper corresponds to that originally suggested by Barndorff‐Nielsen in 1977 [17]. To obtain the parameters that define a theoretical distribution that better fits the empirical data, a maximum likelihood estimation process is used.

## Results

The parameters for each period of the series are shown in Tables 18–24. In those cases, it is possible to fully determine the distribution for each period of the series analyzed. In this case, as the NIG is to be adjusted, the parameter λ = -0.5. Using the Broyden–Fletcher–Goldfarb–Shanno (BFGS) algorithm under maximum likelihood estimation (MLE) criteria, the four parameters left are obtained by this quasi-Newton method. As it is a numeric method, the reduction in parameters decreases the iterations necessary to converge. In addition, as the fixed parameter λ corresponds with the integral K_1_, the convergence conditions are reduced, which saves computational power and time. By comparing the parameter levels, it is possible to observe that for RMB, GBP, HKD and JPY, the second period has the highest alpha levels, meaning a higher concentration of probability around μ. By contrast, EUR and USD have this characteristic in the third period, whereas BRL has it in the first period. The same currency groups present another similarity in the beta parameter. For RMB, GBP, HKD and JPY, the skew level goes from a low positive to high negative and to a lower negative for periods 1, 2 and 3, respectively. In the USD and EUR data, it goes from positive, to negative and to a higher negative, whereas BRL sees a positive, a lower positive and a negative. These results may indicate a disparity and asymmetry in the bubble episodes analyzed.

**Table 18:** Adjusted parameters for BTC/RMB.

Period	𝛍	𝛅	𝛂	𝛃
**1**	0.002014	0.018637	12.453570	0.398361
**2**	0.007116	0.026563	19.254337	-2.723095
**3**	0.007955	0.042780	12.133120	-0.975289

The results were obtained under the maximum likelihood criteria and elaborated by the authors

**Table 19:** Adjusted parameters for BTC/GBP.

Period	𝛍	𝛅	𝛂	𝛃
**1**	0.001289	0.018453	11.454544	0.805273
**2**	0.006072	0.027322	18.742219	-1.644867
**3**	0.008943	0.045662	13.526331	-1.290961

The results were obtained under the maximum likelihood criteria and elaborated by the authors

**Table 20:** Adjusted parameters for BTC/HKD.

Period	𝛍	𝛅	𝛂	𝛃
**1**	0.001451	0.016700	9.654512	0.556296
**2**	0.006489	0.028528	20.418244	-2.365400
**3**	0.008035	0.045188	12.620969	-0.914868

The results were obtained under the maximum likelihood criteria and elaborated by the authors

**Table 21:** Adjusted parameters for BTC/JPY.

Period	𝛍	𝛅	𝛂	𝛃
**1**	1.427e-04	1.815e-02	1.145e+01	1.202e+00
**2**	0.006833	0.029918	22.188326	-2.711315
**3**	0.006652	0.045708	12.518270	-0.538190

The results were obtained under the maximum likelihood criteria and elaborated by the authors

**Table 22:** Adjusted parameters for BTC/BRL.

Period	𝛍	𝛅	𝛂	𝛃
**1**	0.000812	0.016933	26.921302	2.641753
**2**	0.001931	0.015056	15.340180	0.834095
**3**	0.005461	0.032428	11.449760	-0.156720

The results were obtained under the maximum likelihood criteria and elaborated by the authors

**Table 23:** Adjusted parameters for BTC/EUR.

Period	𝛍	𝛅	𝛂	𝛃
**1**	0.000829	0.014672	19.631351	1.589035
**2**	0.004085	0.010659	7.431610	-0.403312
**3**	0.01146	0.05816	20.70262	-2.48932

The results were obtained under the maximum likelihood criteria and elaborated by the authors

**Table 24:** Adjusted parameters for BTC/USD.

Period	𝛍	𝛅	𝛂	𝛃
**1**	0.001546	0.012471	10.117459	0.369375
**2**	0.00409	0.01349	12.15199	-0.34794
**3**	0.01080	0.06645	21.42828	-2.03816

The results were obtained under the maximum likelihood criteria and elaborated by the authors

To prove that the theoretical distribution fits the observed data, it is possible to employ goodness-of-fit criteria, similar to the non-normality test. To do so, a new series was simulated for each period using the parameters previously obtained. Using the nonparametric criteria, the Anderson-Darling, Kolmogorov-Smirnov and Kruskal-Wallis statistics were obtained. In this case, the null hypothesis was that the samples came from the same theoretical distribution. Using the p-values of these statistics, it was possible to not reject the null hypothesis, so the NIG parameters were able to model the observed returns of BTC for all seven series; the results are presented in Tables 25–31.

**Table 25:** Goodness-of-fit test for BTC/RMB.

Period	Anderson-Darling	Kolmogorov-Smirnov	Kruskal-Wallis
**1**	0.6567	0.5795	0.7894
**2**	0.7144	0.787	0.3830
**3**	0.7879	0.5474	0.5781

The p-values of the statistics to prove goodness of fit, elaborated by the authors.

**Table 26:** Goodness-of-fit test for BTC/GBP.

Period	Anderson-Darling	Kolmogorov-Smirnov	Kruskal-Wallis
**1**	0.6431	0.5161	0.3969
**2**	0.5075	0.5176	0.455
**3**	0.4664	0.4895	0.4470

The p-values of the statistics to prove goodness of fit, elaborated by the authors

**Table 27:** Goodness-of-fit test for BTC/HKD.

Period	Anderson-Darling	Kolmogorov-Smirnov	Kruskal-Wallis
**1**	0.7562	0.9278	0.5250
**2**	0.8202	0.9408	0.6393
**3**	0.9301	0.9914	0.7303

The p-values of the statistics to prove goodness of fit, elaborated by the authors

**Table 28:** Goodness-of-fit test for BTC/JPY.

Period	Anderson-Darling	Kolmogorov-Smirnov	Kruskal-Wallis
**1**	0.8070	0.7736	0.7310
**2**	0.6682	0.7212	0.4737
**3**	0.9252	0.9793	0.84880

The p-values of the statistics to prove goodness of fit, elaborated by the authors

**Table 29:** Goodness-of-fit test for BTC/BRL.

Period	Anderson-Darling	Kolmogorov-Smirnov	Kruskal-Wallis
**1**	0.7281	0.9278	0.7215
**2**	0.7776	0.6527	0.4252
**3**	0.5045	0.6074	0.4208

The p-values of the statistics to prove goodness of fit, elaborated by the authors

**Table 30:** Goodness-of-fit test for BTC/EUR.

Period	Anderson-Darling	Kolmogorov-Smirnov	Kruskal-Wallis
**1**	0.9954	0.9812	0.7329
**2**	0.2013	0.2923	0.4068
**3**	0.9688	0.9914	0.7316

The p-values of the statistics to prove goodness of fit, elaborated by the authors

**Table 31:** Goodness-of-fit test for BTC/USD.

Period	Anderson-Darling	Kolmogorov-Smirnov	Kruskal-Wallis
**1**	0.5692	0.5795	0.3419
**2**	0.8116	0.8996	9.989e-01
**3**	0.9255	0.7875	0.87840

The p-values of the statistics to prove goodness of fit, elaborated by the authors

Once it was proven that NIG is able to fit the empirical return distribution for all of the BTC exchange rates, even capturing explosive behavior, it could be used to estimate the VaR and CVaR. Using the third period as the reference point, the values for VaR and CVaR for the distributions at a 95% confidence level are presented in Tables 32 and 33. The results indicate that the VaR level for NIG is smaller than that for the GH; nevertheless, this is a millesimal difference in return levels. However, as stated in the literature [44], the CVaR is a better approximation for studying risk exposure because the expected losses surpass the VaR level. For these particular cases, the expected shortfall obtained with the NIG is consistently larger than that obtained with the GH, as would be expected with a lower VaR. Again, this indicates only a marginal improvement from using the GH relative to the NIG.

**Table 32:** VaR 95%.

Distribution	RMB	GBP	HKD	JPY	BRL	EUR	USD
**NIG**	0.09	0.08943	0.090683	0.090602	0.07548	0.084454	0.08811
**GH**	0.0915	0.0928	0.0914	0.09159	0.07734	0.0853	0.08913

VaR levels according to theoretical distribution in absolute values, elaborated by the authors

**Table 33:** CVaR 95%.

Distribution	RMB	GBP	HKD	JPY	BRL	EUR	USD
**NIG**	0.14274	0.13948	0.14213	0.14111	0.12391	0.1238	0.1267
**GH**	0.14123	0.13677	0.14148	0.14012	0.12027	0.12326	0.1257

CVaR levels according to theoretical distribution in absolute values, elaborated by the authors

**Fig 1:**
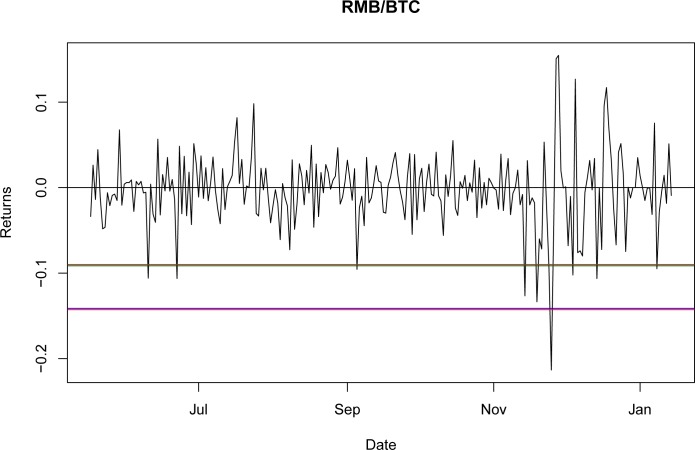
VaR and CVaR levels RMB/BTC. Returns for out-of-sample data, estimated VaR and CVaR with NIG and GH distributions. Blue: VaR GH, Red: VaR NIG, Green: CVaR GH, Brown: CVaR NIG. Elaborated by authors.

**Fig 2:**
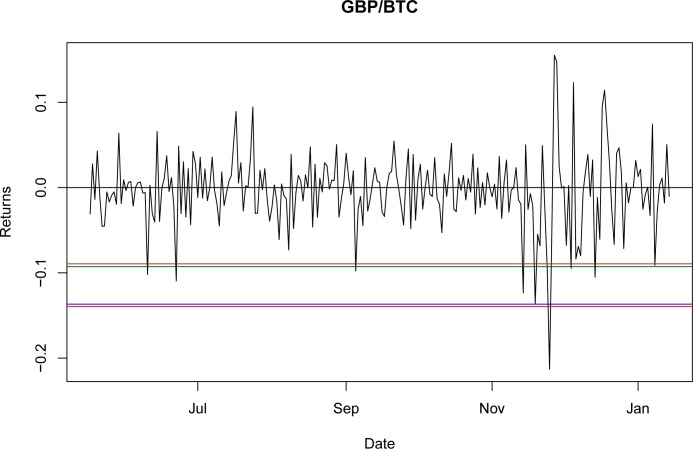
VaR and CVaR levels GBP/BTC. Returns for out-of-sample data, estimated VaR and CVaR with NIG and GH distributions. Blue: VaR GH, Red: VaR NIG, Green: CVaR GH, Brown: CVaR NIG. Elaborated by authors.

**Fig 3:**
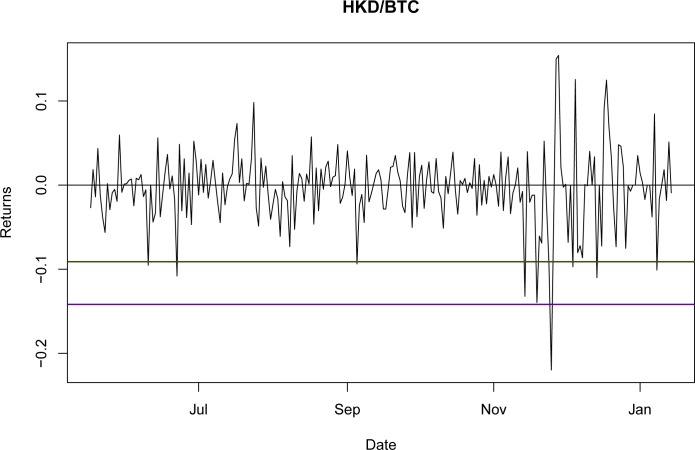
VaR and CVaR levels HKD/BTC. Returns for out-of-sample data, estimated VaR and CVaR with NIG and GH distributions. Blue: VaR GH, Red: VaR NIG, Green: CVaR GH, Brown: CVaR NIG. Elaborated by authors.

**Fig 4:**
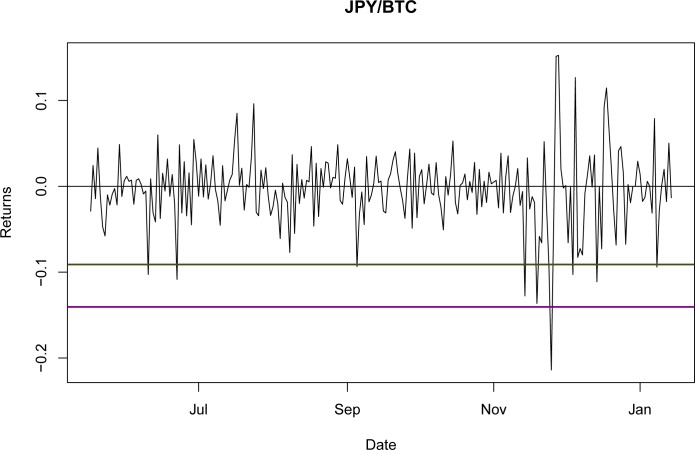
VaR and CVaR levels JPY/BTC. Returns for out-of-sample data, estimated VaR and CVaR with NIG and GH distributions. Blue: VaR GH, Red: VaR NIG, Green: CVaR GH, Brown: CVaR NIG. Elaborated by authors.

**Fig 5:**
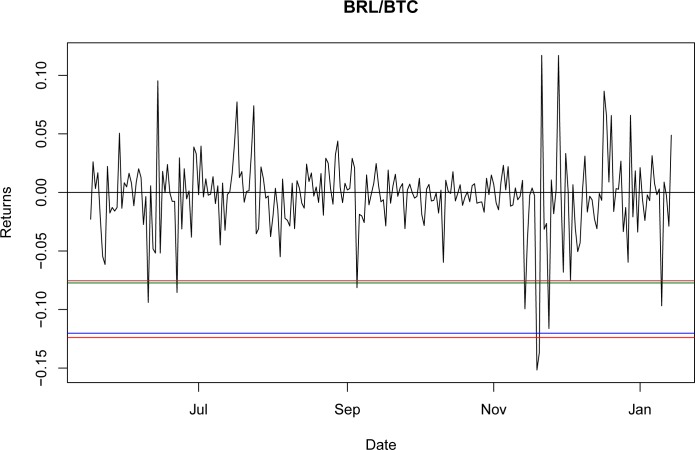
VaR and CVaR levels BRL/BTC. Returns for out-of-sample data, estimated VaR and CVaR with NIG and GH distributions. Blue: VaR GH, Red: VaR NIG, Green: CVaR GH, Brown: CVaR NIG. Elaborated by authors.

**Fig 6:**
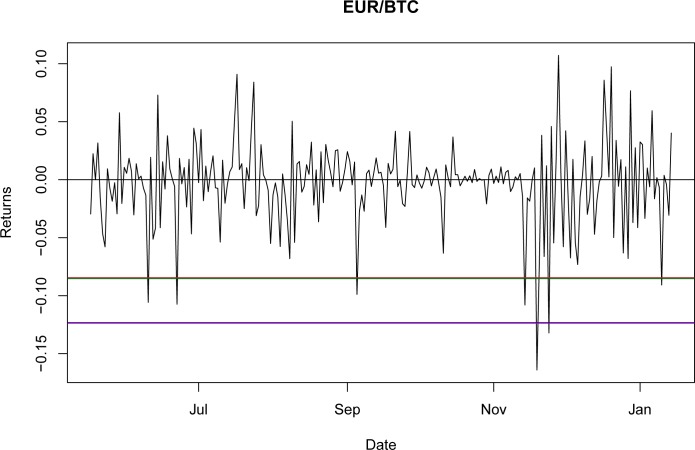
VaR and CVaR levels EUR/BTC. Returns for out-of-sample data, estimated VaR and CVaR with NIG and GH distributions. Blue: VaR GH, Red: VaR NIG, Green: CVaR GH, Brown: CVaR NIG. Elaborated by authors.

**Fig 7:**
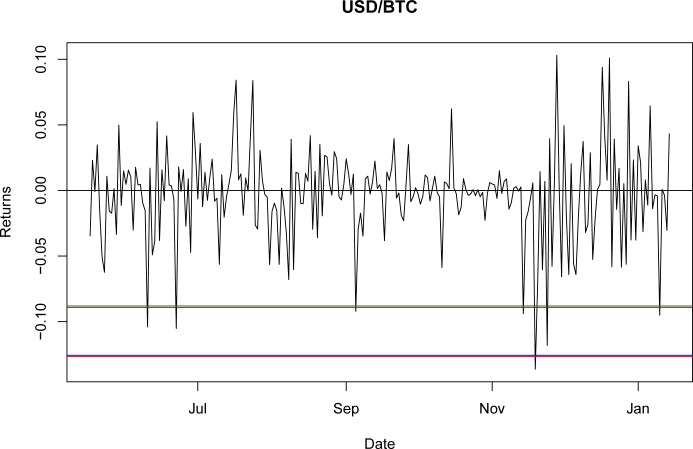
VaR and CVaR levels USD/BTC. Returns for out-of-sample data, estimated VaR and CVaR with NIG and GH distributions. Blue: VaR GH, Red: VaR NIG, Green: CVaR GH, Brown: CVaR NIG. Elaborated by authors.

A graphical representation of these levels for the seven currencies is presented in Figs 1–7.

## Conclusion

The evolution of technology and its invasion of different aspects of human interactions is indisputable. Perhaps the most notable innovation as well as the riskiest is the development of new currencies backed only by mathematical cryptography and operated through computational devices. These cryptocurrencies are a new entity that do not fit into any theoretical framework. This lack of full understanding and a tendency toward self-fulfilling prophesies have led to their explosive behavior since their creation. Furthermore, the speculative factor operating behind them has also led to financial bubbles.

In this paper, the returns of the most-traded cryptocurrency were analyzed in comparison to seven major exchange rates to adjust a theoretical distribution for different periods in which bubble behavior has been detected. The candidate distribution is a member of the hyperbolic family denominated the NIG. This distribution has multiple properties that are useful in the finance field, such as being well adjusted to heavy tails, flexible enough to adapt to skew and kurtosis and close under convolution. By dividing the data into different periods, it was possible to obtain the particular parameters for each time segment. Using statistical tests, we could confirm that the NIG manages to fit these time segments for the cases of RMB, GBP, HKD, JPY, BRL, EUR and UDS data. For further comparison, out-of-sample VaR and CVaR were obtained with NIG and GH distributions. These results show only marginal differences, with the NIG having the higher cumulative density for the expected shortfall. This result coincides with the ability of NIG to model heavy-tailed behavior, such as found in multiple studies regarding BTC.

Finally, these tests have justified the employment of NIG as a better, or at least equivalent, candidate distribution in comparison to GH that is able to model bubble episodes and demonstrate outstanding performance in out-of-sample VaR and CVaR. In addition, as stated earlier, NIG has fewer parameters to adjust and desirable mathematical properties that can be exploited in future works.
